# Supplementary material for: A cell-autonomous tumour suppressor role of RAF1 in hepatocarcinogenesis
Source: Nat Commun. 2016 Dec 21;7:13781. doi: 10.1038/ncomms13781 (PMC5187498; doi:10.1038/ncomms13781)
Supplement: Supplementary Information — Supplementary Figures and Supplementary Tables. [file ncomms13781-s1.pdf]

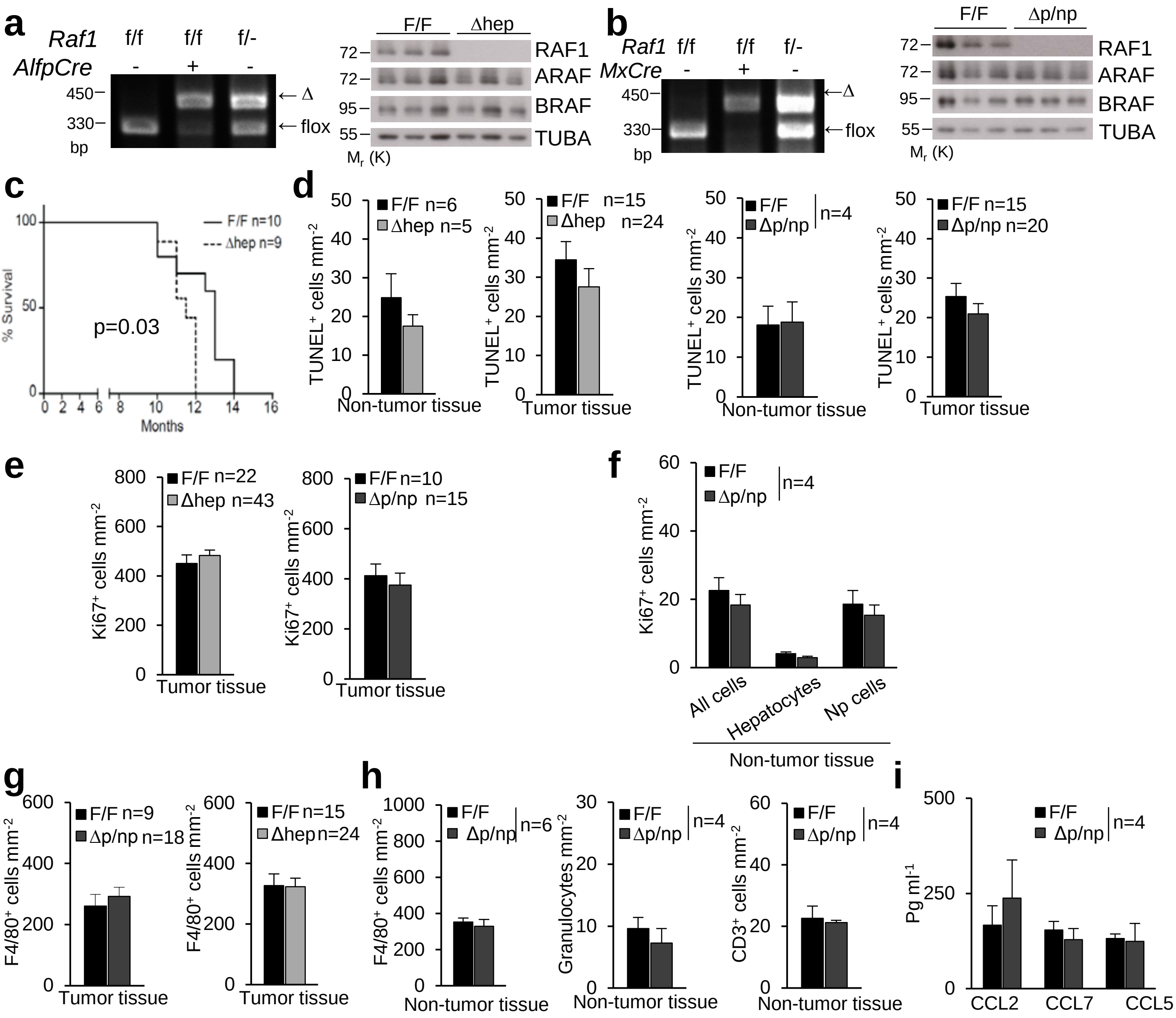

k

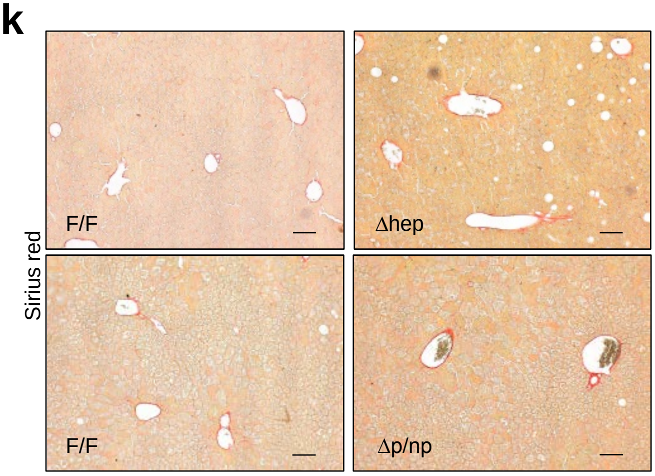

j

Chemo-/cytokines in tumor-bearing livers (pg mg-1 protein)	F/F (n=3)	Δp/np (n=3)
CCL2	1,037 (+/-74)	1,136 (+/-59)
CCL4	322 (+/-34)	380 (+/-28)
CCL5	334 (+/-45)	400 (+/-50)
CCL7	65 (+/-10)	77 (+/-10)
GMCSF	190 (+/-23)	219(+/-11)
CXCL1	85 (+/-7)	99 (+/-12)
IL2	505 (+/-55)	546 (+/-43)
IL4	187 (+/-40)	192 (+/-11)
IL5	620 (+/-80)	647 (+/-33)
IL6	460 (+/-40)	519(+/- 34)
IL10	400 (+/-85)	423 (+/-34)
IL27	247 (+/-77)	264 (+/-15)
TNFα	365 (+/-93)	386 (+/-30)
IL1β	1,050 (+/-206)	1,127 (+/-83)
IFNγ	386 (+/-90)	415 (+/-37)
IL17	386 (+/-95)	400 (+/-24)
IL1α	224 (+/-23)	222 (+/-12)
IL13	224 (+/-56)	244 (+/-15)

Supplementary Figure 1. RAF1 ablation promotes chemical hepatocarcinogenesis.

a-b, PCR and immunoblotting analysis of liver samples isolated from 4 weeks (w) old animals of different genotypes. Note the conversion of the F to the Δ band, the absence of RAF1 expression and the unperturbed ARAF and BRAF expression in the *AlfpCre;Raf*$^{F/F}$ (**a**) and *MxCre;Raf1*$^{F/F}$ (**b**) livers. Tubulin (TUBA) serves as a loading control. **c,** Kaplan-Meier plot depicting the survival of DEN/Pb-treated F/F and Δhep mice. The data were analyzed using the log-rank test. $\chi 2=4.672$, $p=0.03$. **d,** similar numbers of apoptotic cells (TUNEL$^+$) in F/F, Δhep and Δp/np tumor-bearing livers. **e**, comparable numbers of cycling cells (Ki67$^+$) in F/F, Δhep and Δp/np tumors. **f**, comparable amounts of cycling cells (Ki67$^+$) in F/F and Δp/np non-tumor tissue **g**, comparable amounts of F4/80$^+$ cells in tumors of F/F, Δhep and Δp/np mice and (**h**) similar numbers of F4/80$^+$ cells, granulocytes and CD3$^+$ lymphocytes in F/F and Δp/np non-tumor tissue. **i**, serum chemokine levels in F/F and Δp/np mice. **j**, comparable amounts of chemo- and cytokines in the lysates of F/F and Δp/np tumor-bearing livers. **k**, no evidence of fibrosis in F/F, Δhep and Δp/np tumor-bearing livers (representative image of a Sirius red-stained section, n=5 per genotype). Scale bar, 50 µm. Data are represented as mean ± SEM. Unless otherwise stated, all organs were analyzed 30 w after DEN/Pb treatment.

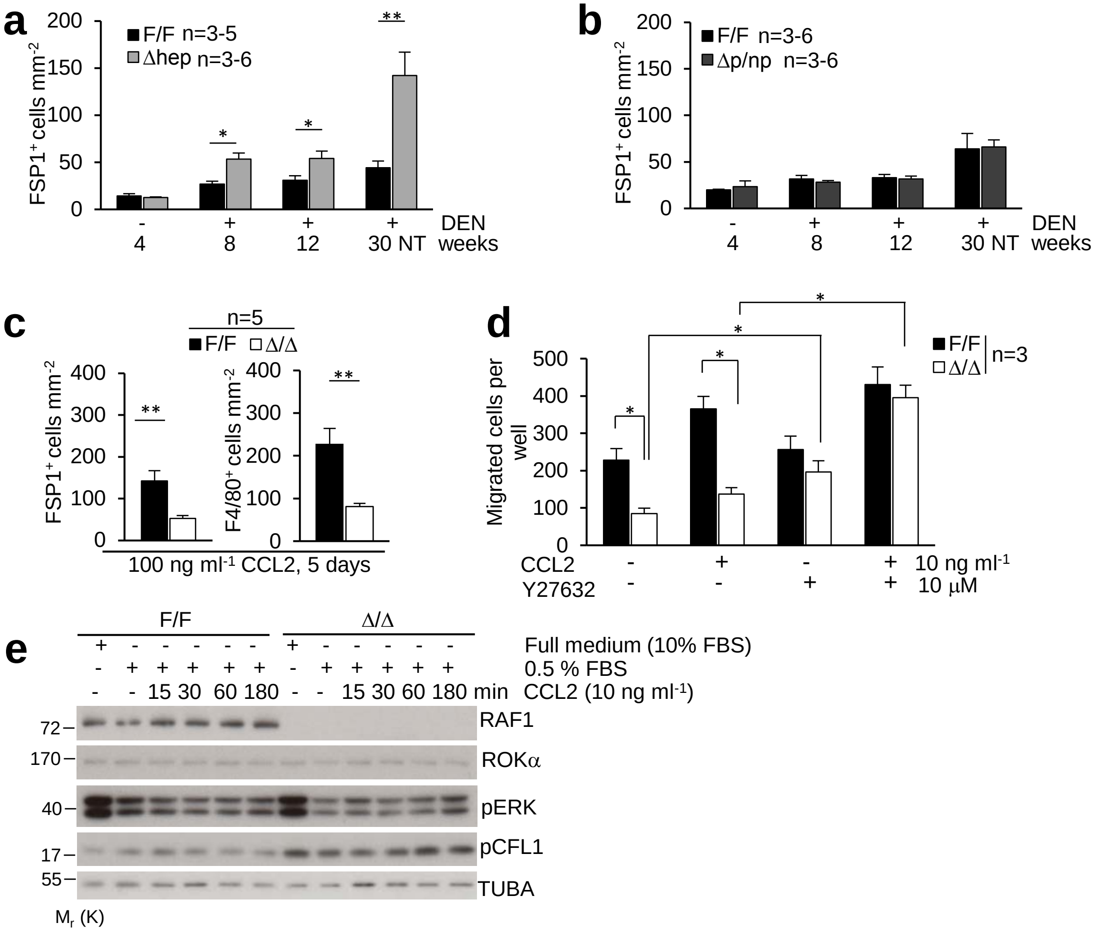

Supplementary Figure 2. RAF1-deficient macrophages fail to accumulate in tumor-bearing livers and to migrate towards CCL2 *in vivo* and *in vitro*.

a-b, FSP1$^+$ non-parenchymal cells at different stages of tumorigenesis in the non-tumor tissue of Δhep (**a**) and Δp/np livers (**b**). **c-d,** migration defects of RAF1-deficient macrophages. (**c**), FSP1$^+$ and F4/80$^+$ cells migrated in CCL2-supplemented matrigel plugs implanted in F/F and Δp/np mice. **d,** F/F and Δ/Δ bone marrow derived macrophages (BMDM), untreated or pre-treated for 30 min with Y27632, were allowed to migrate towards CCL2 for 6 hours (h). **e,** increased ROKα signaling in Δ/Δ BMDM. F/F and Δ/Δ BMDM were treated with CCL2 for the indicated times prior to lysis and immunoblotting. CFL1, cofilin; TUBA serves as loading control. Data represent the mean ± SEM. *p≤0.05, **p<0.01, ***p<0.005 according to Student's t test. See also Supplementary Table 1.

FSP1$^+$ cells **a**: 4 w no DEN (n=3 per genotype), 8 w DEN (n=4 per genotype), 12 w DEN (F/F, n=4; Δhep, n=5), 30 w DEN NT (F/F, n=5; Δhep, n=6); **b**: 4 w no DEN (n=3 per genotype), 8 w DEN (F/F, n=4; Δp/np, n=5), 12 w DEN (F/F, n=5; Δp/np, n=4), 30 w DEN NT (n=6 per genotype).

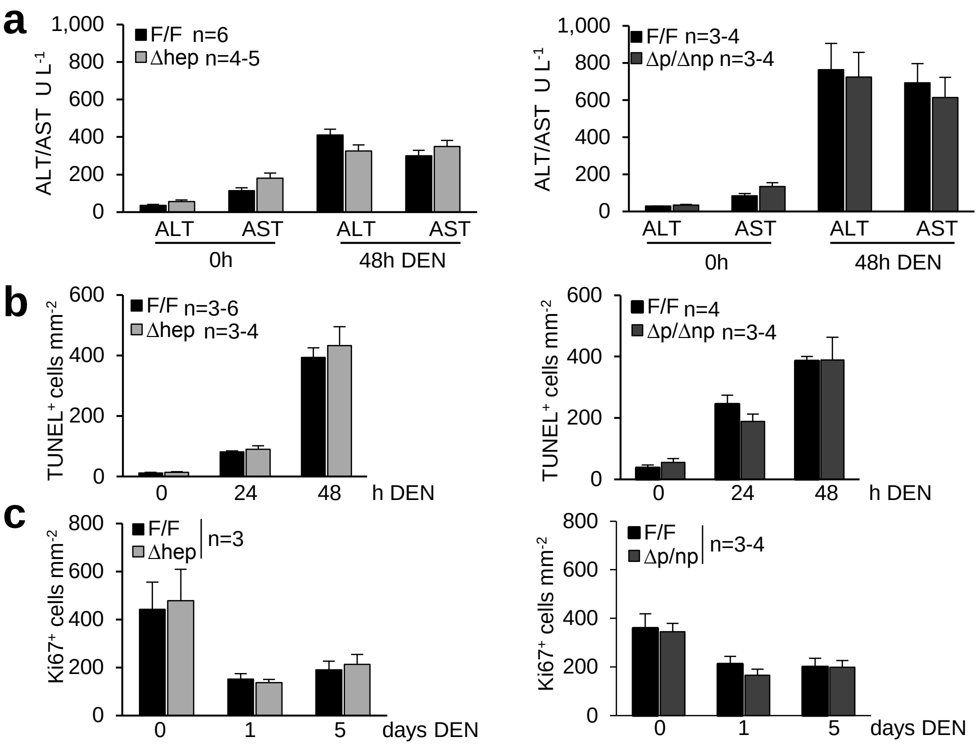

Supplementary Figure 3. Impact of RAF1 ablation on the early phase of DEN-induced liver damage and regeneration.

Similar ALT/AST serum concentrations (**a**) liver apoptotic cells (TUNEL$^+$, **b**) and cycling cells (Ki67$^+$, **c**) in DEN-treated Δhep and Δp/np mice, each compared with their lttermate controls. Data are presented as mean ± SEM.

a: ALT/AST serum levels: 0h (Δhep n=4, F/F n=6) 48h DEN (Δhep n=5, F/F n=6); right panel 0h (n=3 per genotype) 48h DEN (n=4 per genotype). **b**: TUNEL$^+$ cells, 0h (Δhep n=3, F/F n=4), 24h DEN (Δhep n=4, F/F n=3), 48h DEN (Δhep n=4, F/F n=6): right panel 0h (Δp/np n=3, F/F n=4), 24h and 48h DEN (n=4 per genotype). **c**, Ki67$^+$ cells, all timepoints n=3 per genotype; right panel 0 no DEN (Δp/np n=3, F/F n=4), 1 day DEN (n=3 per genotype), 5 days DEN (n=4 per genotype).

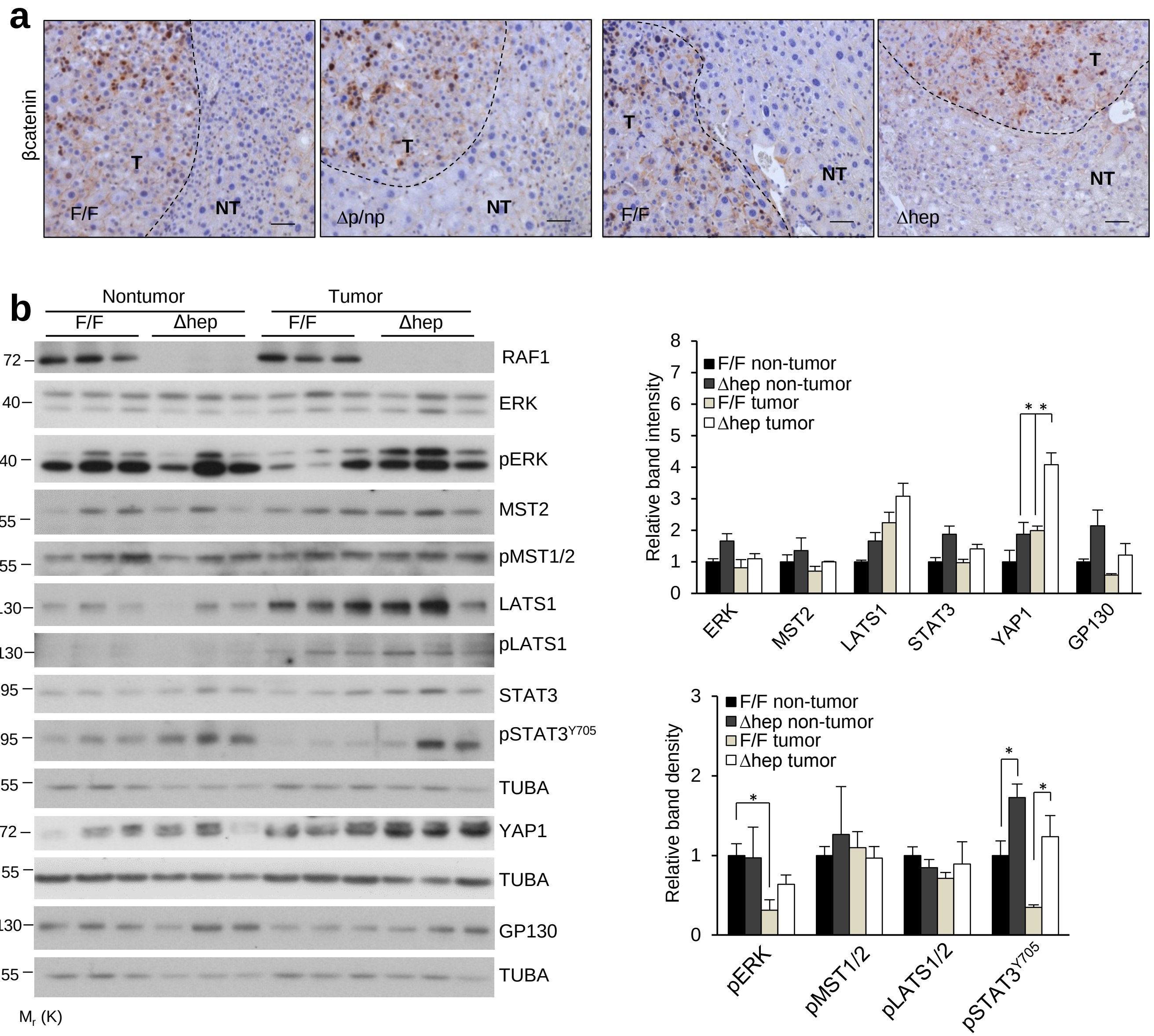

Supplementary Figure 4. Molecular characterization of RAF1-deficient lesions and cells.
a, RAF1 ablation does not affect βcatenin expression or localization (IHC images representative of 3 mice). Scale bar, 50 µm. **b,** effect of hepatocyte-restricted RAF1 ablation on signaling pathways in tumor-bearing livers. All organs were isolated 30 w after DEN injection. The plots represent a densitometric quantification of the immunoblots performed using ImageJ. The data are expressed as relative band density adjusted to TUBA, which serves as loading control (upper plot). Phosphorylation (lower plot) is expressed as the ratio between the signal obtained with the phosphospecific antibodies and the signal obtained with the protein-specific antibodies. In both cases, the data are normalized to the F/F non-tumor samples, which are arbitrarily set as 1. Data are presented as mean ± SEM, *p≤0.05 according to Student's t test.

a

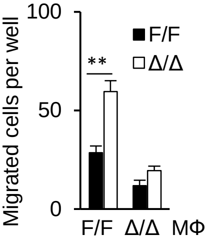

b

DIH	F/F (n=3)			Δ/Δ (n=3)		
Pg mg^{-1} protein	/	10 ng ml^{-1} LPS	1 µg ml^{-1} LPS	/	10 ng ml^{-1} LPS	1 µg ml^{-1} LPS
CCL2	813 (+/-41)	6,990 (+/-222)	5,567 (+/-581)	973 (+/-27)*	8,633 (+/-448)*	8,567 (+/-581)*
CCL7	117 (+/-47)	1,033 (+/-186)	1,400 (+/-600)	40 (+/-12)	672 (+/-160)	705 (+/-190)
CXCL1	953 (+/-200)	8,500 (+/-1,700)	8,233 (+/-906)	1,733 (+/-133)*	18,833 (+/-600)*	16,233 (+/-1130)*

c

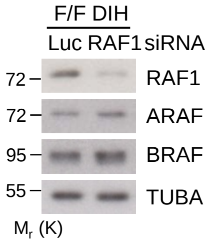

d

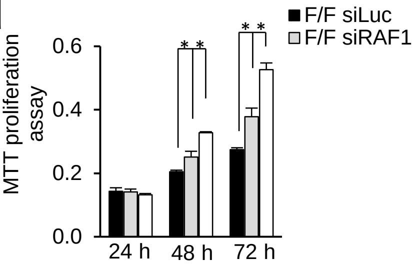

e

DIH	F/F siLuc (n=3)			F/F siRAF1 (n=3)		
Pg mg^{-1} protein	/	10 ng ml^{-1} LPS	1 µg ml^{-1} LPS	/	10 ng ml^{-1} LPS	1 µg ml^{-1} LPS
CCL2	718 (+/-109)	6,347 (+/-52)	5,606 (+/-454)	722 (+/-43)	8,655 (+/-600)*	9,341 (+/-525)*
CCL7	296 (+/-47)	852 (+/-16)	1,019 (+/-191)	0 (+/-0)	774 (+/-141)	913 (+/-125)
CXCL1	777 (+/-239)	6,788 (+/-1,302)	6,021 (+/-1,583)	1,024 (+/-140)	11,832(+/-1,269)*	15,386(+/-1,653)*

f

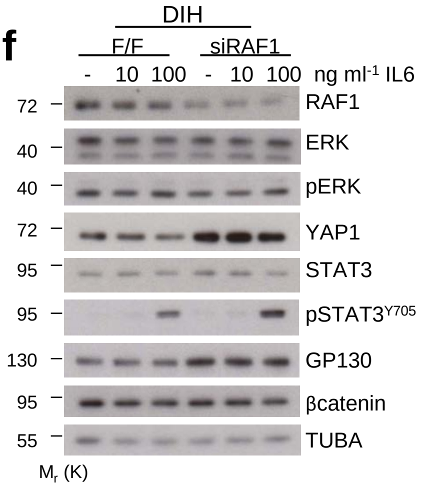

Supplementary Figure 5. Characterization of RAF1-deficient DIH.

a, increased chemotactic activity of Δ/Δ DIH towards wild-type, but not RAF1-deficient bone marrow-derived macrophages (BMDM; MΦ). MΦ were allowed to migrate for 6 h. n=3 independent experiments. **b**, Increased chemokine production of Δ/Δ DIH versus F/F DIH treated with LPS for 24h. **c,** immunoblot analysis of F/F siLuc and F/F siRAF1, note the efficent RAF1 downregulation and the lack of effect on ARAF and BRAF expression. **d**, proliferation of F/F siLuc and F/F siRAF1 DIH. Δ/Δ siLuc DIH are shown as a control. Proliferation in 5% FBS DIH medium was determined using the MTT test. **e**, chemokine profile of F/F siLuc and F/F siRAF1 DIH treated with LPS for 24h. **f**, immunoblot analysis of F/F siLuc and F/F siRAF1 treated with indicated concentrations of IL6 for 30 min. The data represent the mean ± SEM of 3 independent experiments. *p≤0.05, **p<0.01 according to Student's t test.

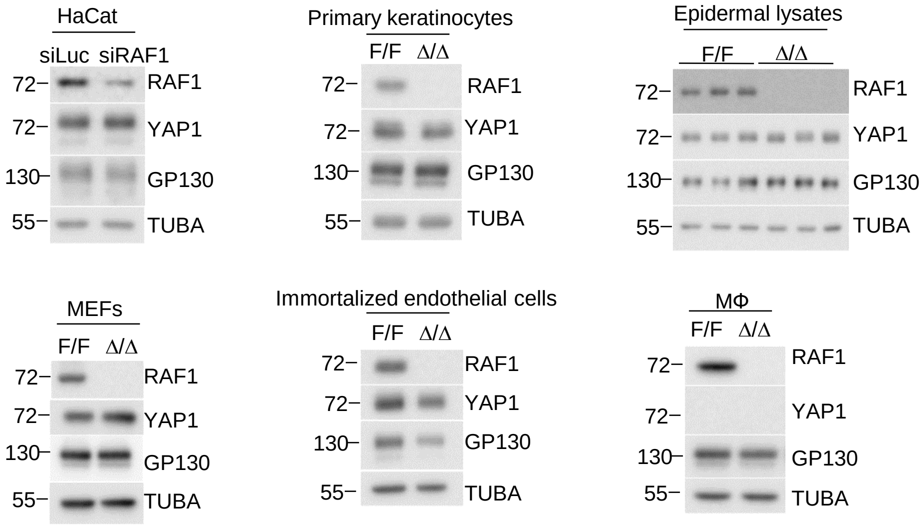

Supplementary Figure 6. YAP1/GP130 expression does not increase in a range of RAF1-deficient cell types.

Expression was analyzed by immunoblotting in the indicated RAF1-proficient and -deficient cells. TUBA is shown as a loading control.

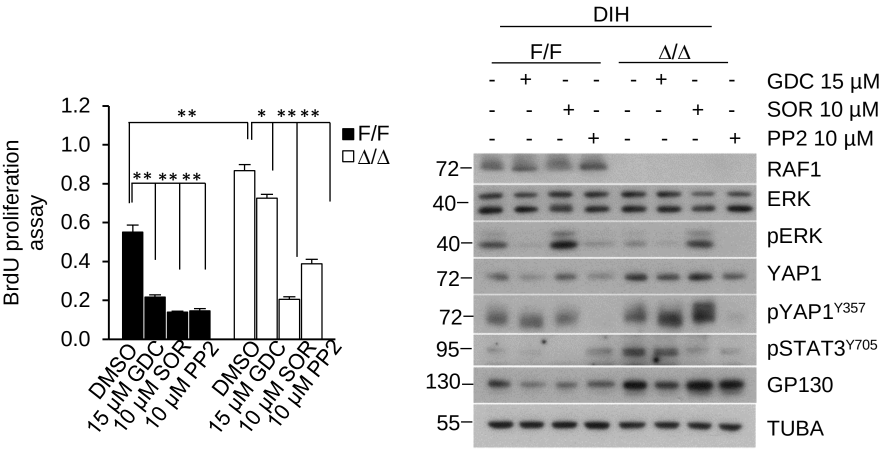

Supplementary Figure 7. Effect of RAF or multikinase inhibitors on RAF1-proficient and -deficient DIH. Left panel, BrdU incorporation and right panel, immunoblot analysis of DIH treated with GDC-0879 (GDC), Sorafenib (SOR) or PP2. Proliferation was assessed after a 48 h treatment, and immunoblotting after 1 h teatment. TUBA, loading control. The data represent the mean ± SEM. *p≤0.05, **p<0.01 according to Student's t test.

Figure 1b

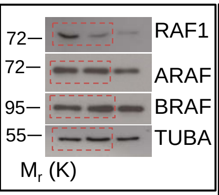

Figure 3a

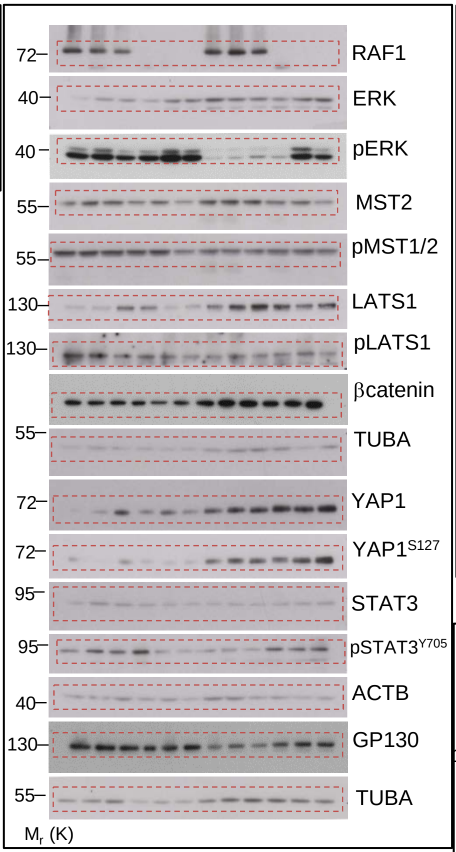

Figure 3b

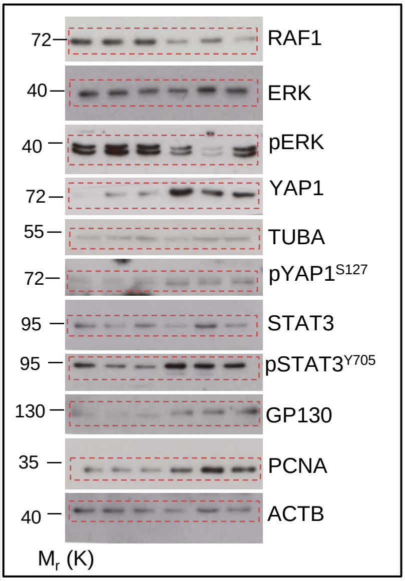

Figure 4a

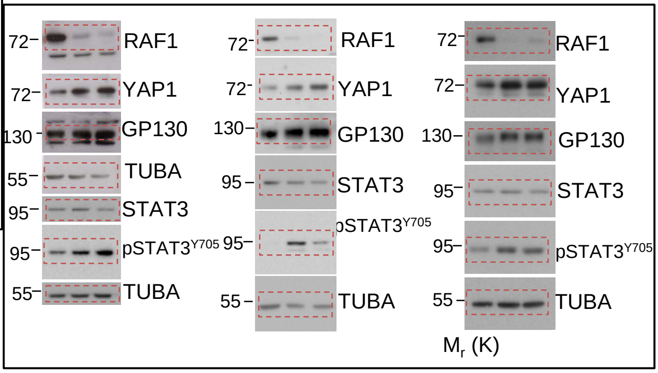

Figure 4b

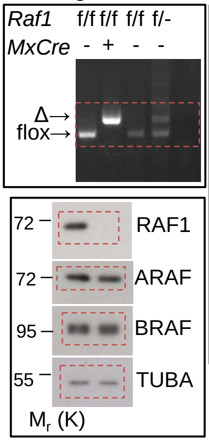

Figure 4d

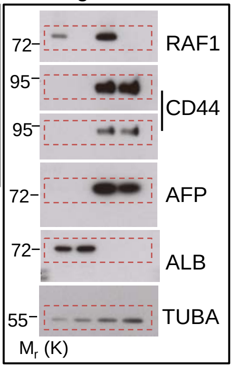

Figure 4f

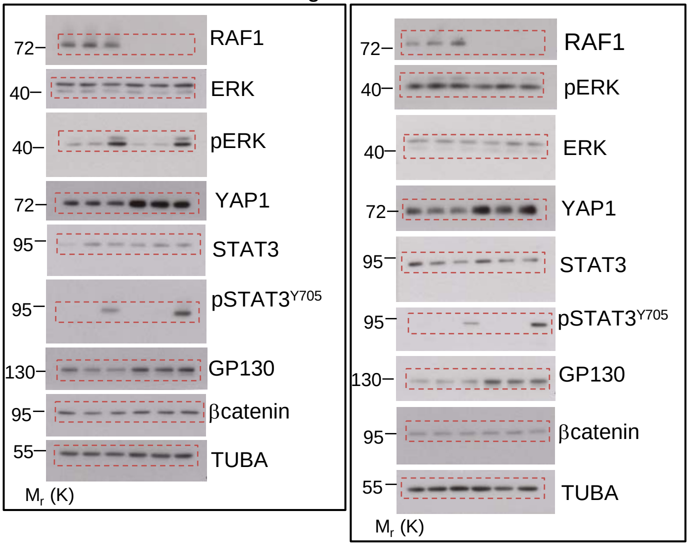

Figure 5a

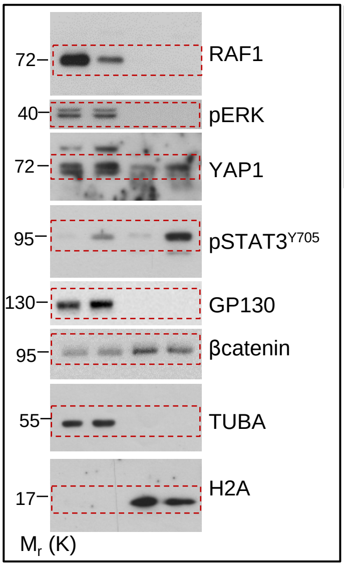

Figure 5b

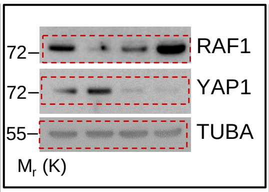

Figure 5c

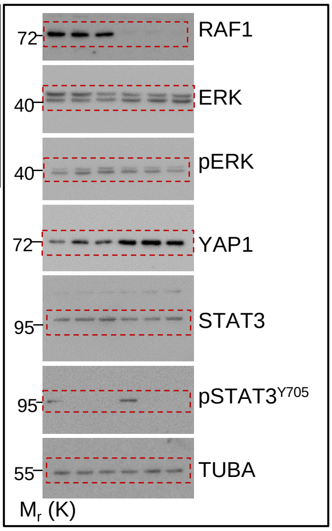

Figure 5d

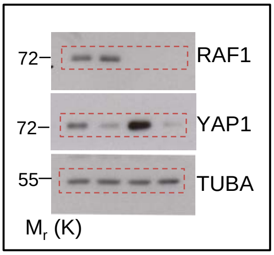

Figure 5e

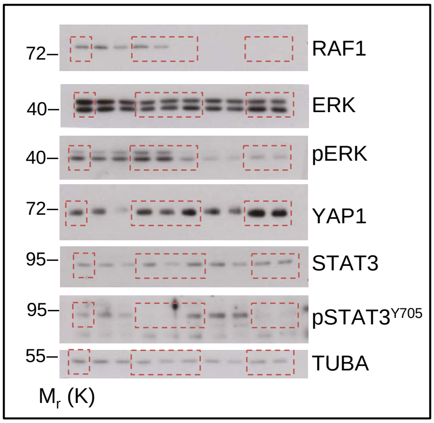

Figure 5f

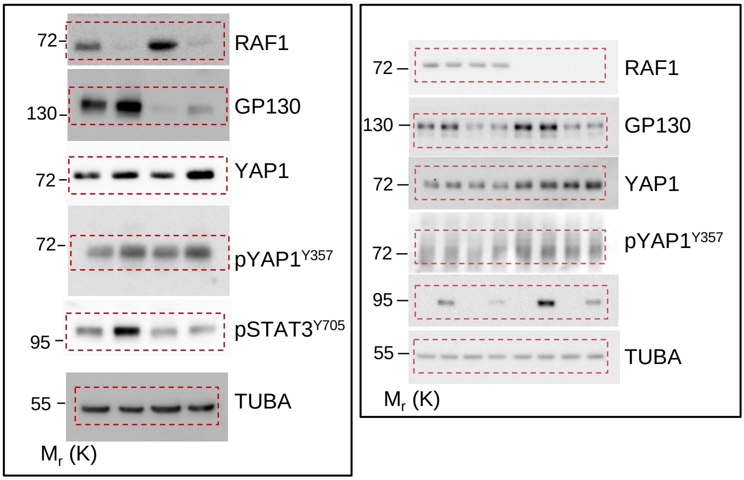

Figure 6d

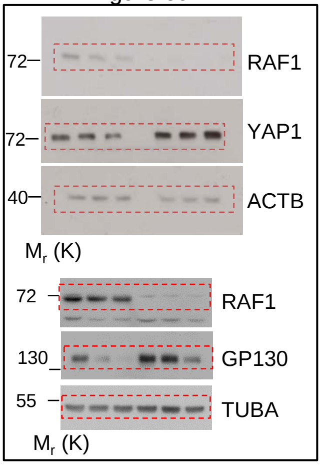

Figure 6e

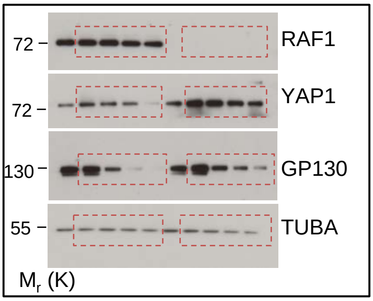

Figure 6f

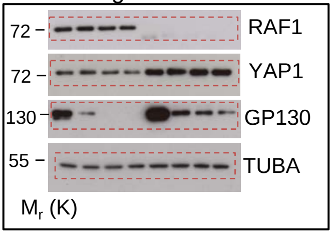

Supplementary Fig. 1a

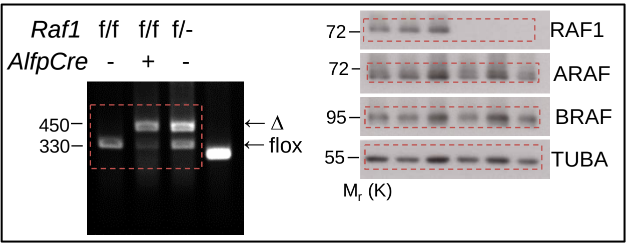

Supplementary Fig. 1b

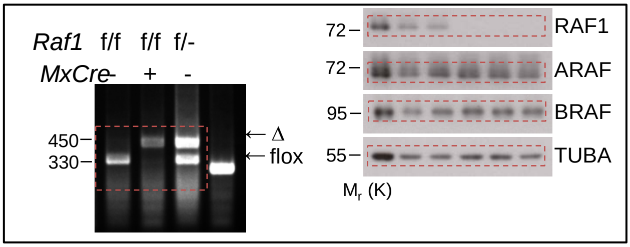

Supplementary Fig. 2a

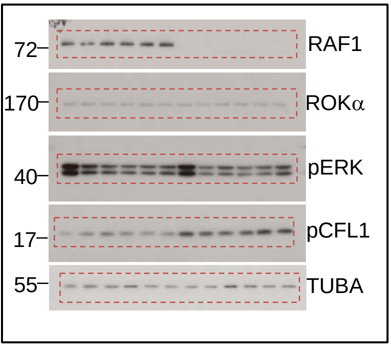

Supplementary Fig. 4b

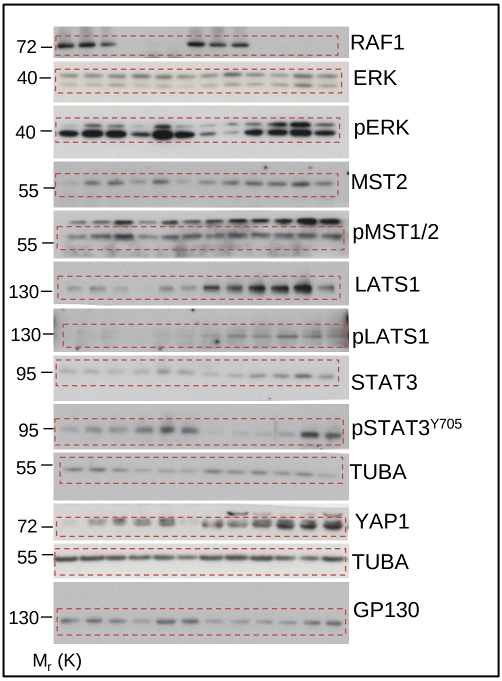

Supplementary Fig. 5c

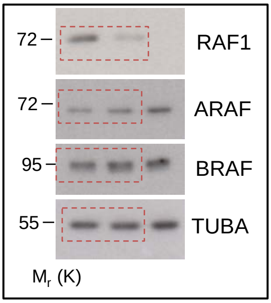

Supplementary Fig. 5f

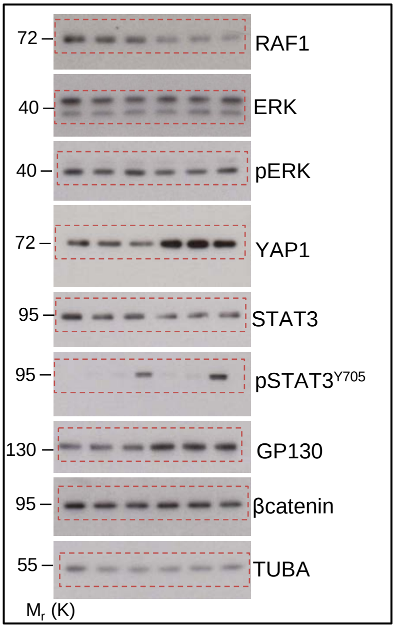

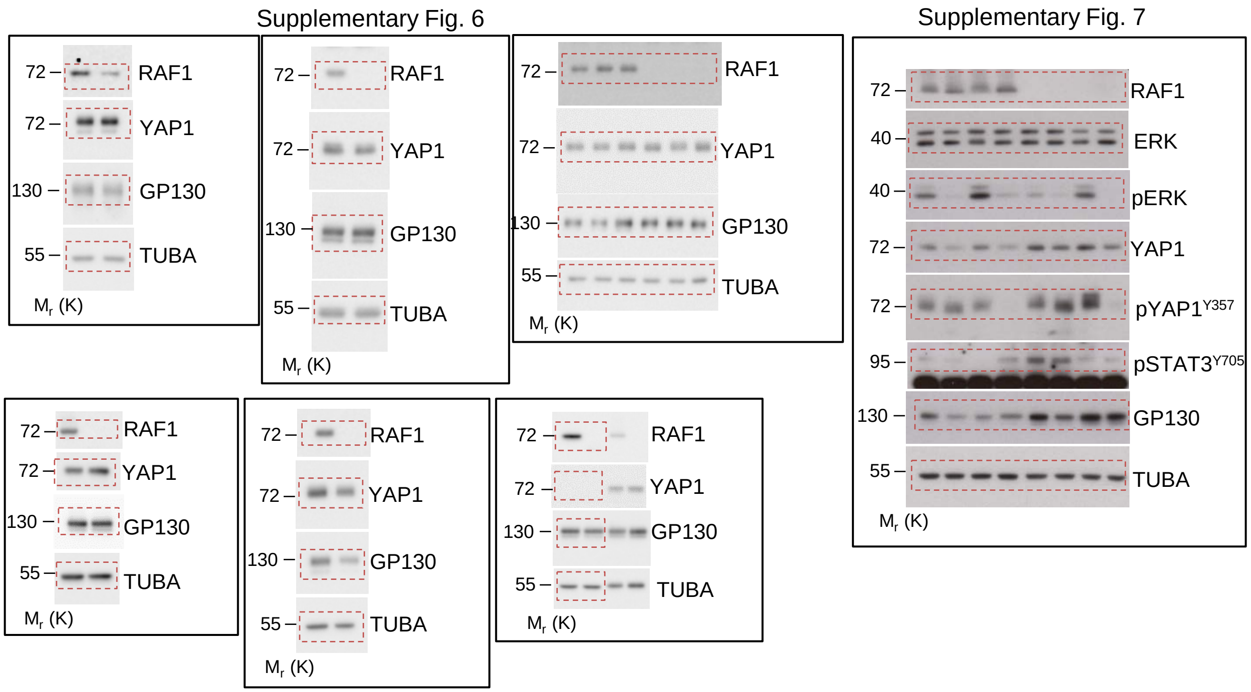

Supplementary Figure 8. Uncropped immunoblots.

Supplementary Table 1. Chemokine production by F/F and RAF1-deficient cells. F/F and Δ/Δ P-HEPS and BMDMs were stimulated with the indicated concentrations of LPS for 24 h. The results are expressed as mean ± SEM, *p<0.05 according to Student's t test.

P-HEPS (n=4)	F/F			Δ/Δ		
Pg mg^{-1} protein	/	10 ng ml^{-1} LPS	1 µg ml^{-1} LPS	/	10 ng ml^{-1} LPS	1 µg ml^{-1} LPS
CCL2	2,030 (+/-424)	10,509 (+/-1,600)	13,727 (+/-1,730)	3,895 (+/-569)*	13,115 (+/-2,346)	19,106 (+/-579)*
CCL7	4 (+/-0)	84 (+/-18)	151 (+/-36)	5 (+/-0)	113 (+/-32)	185 (+/-62)
CXCL1	320 (+/-100)	717 (+/-110)	929 (+/-140)	320 (+/-100)	731 (+/-150)	1,110 (+/-200)
BMDMs (n=6)	F/F			Δ/Δ		
Pg mg^{-1} protein	/	10 ng ml^{-1} LPS	1 µg ml^{-1} LPS	/	10 ng ml^{-1} LPS	1 µg ml^{-1} LPS
CCL2	0 (+/-0)	22,341 (+/-5,240)	32,285 (+/-8,300)	0 (+/-0)	22,002 (+/-2,188)	24,364 (+/-3,120)
CCL7	0 (+/-0)	3,990 (+/-400)	5,230 (+/-474)	0 (+/-0)	4,752 (+/-990)	4,950 (+/-900)
CXCL1	187 (+/-25)	18,544 (+/-3,170)	18,305 (+/-2,817)	193 (+/-46)	18,983 (+/-3,300)	19,229 (+/-3,100)
IL6	0 (+/-0)	13,401 (+/-3,390)	22,041 (+/-4,131)	0 (+/-0)	18,532 (+/-4,809)	29,199 (+/-6,341)
TNFα	0 (+/-0)	18,857 (+/-6,500)	22,000 (+/-5,400)	0 (+/-0)	24,000 (+/-7,200)	32,600 (+/-8,900)
IL1β	0 (+/-0)	1,400 (+/-248)	3,514 (+/-237)	0 (+/0)	828 (+/-200)	2,650 (+/-100)

Supplementary Table 2. Correlation between clinical parameters, RAF1 and YAP1 expression in human HCC. RAF1 and YAP1 expression in tumor (defined as the ratio of RAF1 or YAP1 expression in matched tumor/non-tumor tissues) is divided in 3 groups (low, <0.80; similar, 0.80-1.20; and high, >1.20).

			GENDER		CIRRHOSIS		SURVIVAL (months)	
Protein expression in tumors			m	f	no	yes	<42	>42
RAF1 low (n =16)	YAP1	high (n=10)	7	3	7	3	5	2
		similar (n=3)	3			3	3	
		low (n=3)	3		1	2	2	1
RAF1 similar (n=11)	YAP1	high (n=3)	3		2	1	2	1
		similar (n=5)	5		3	2	1	3
		low (n=3)	3			3		2
RAF1 high (n=4)	YAP1	high (n=1)		1		1	1	
		similar (n=2)	2		2		1	1
		low (n=1)	1		1			1
Subtotal			27	4	16	15	15	11
Total			31		31		26[a]	

[a] 5 HCC-unrelated death causes were not included in the survival rate calculation. No significant correlation was observed between the expression levels of YAP1 or RAF1 and the survival rate.
